# Linking platelet indices and uterine artery Doppler parameters for preeclampsia prediction and its severity

**DOI:** 10.6026/9732063002001829

**Published:** 2024-12-31

**Authors:** Simran Verma, Shikha Singh, Nilam Bhasker, Meenal Jain, Sangita Sahu, Hari Singh, Navya Verma

**Affiliations:** 1 Department of Obstetrics and Gynaecology, All India Institute of Medical Sciences Raebareli, Raebareli, Uttar Pradesh, India; 2 Department of Obstetrics and Gynaecology, S.N. Medical College, Agra, Uttar Pradesh, India; 3 Department of Pathology, ESIC Hospital, Lucknow, Uttar Pradesh, India; 4 Department of Obstetrics and Gynaecology, S.N. Medical College, Agra, Uttar Pradesh, India; 5 Department of Radiodiagnosis, S.N. Medical College, Agra, Uttar Pradesh, India; 6 Modern School, Lucknow, Uttar Pradesh, India

**Keywords:** Platelet count, mean platelet volume, platelet distribution width, plateletcrit, platelet/lymphocyte ratio, uterine artery pulsatility index, resistivity index, systolic/diastolic ratio, preeclampsia

## Abstract

Preeclampsia is one of the most common medical complications of pregnancy and the second largest cause of maternal and fetal mortality and morbidity. Preeclampsia is a multi-systemic hypertension condition with an unknown cause. A Prospective Observational Study was conducted on antenatal women with gestation age of 11 to 13+6 weeks. Participants underwent routine investigations including Platelet Indices and Uterine Artery Ultrasonography Doppler. Selected patients were specifically evaluated at 22-24 weeks, 31-34 weeks and >36 weeks until delivery along with routine antenatal care visits. Among the platelet indices, the platelet Count, Mean Platelet volume (MPV), Platelet/Lymphocyte ratio were found significant and among Uterine Artery Doppler Parameters- Pulsatility Index was found significant in prediction of preeclampsia. The Combination of platelet indices and uterine artery doppler parameters showed better sensitivity in prediction of early onset and severe form of preeclampsia can be incorporated into routine prenatal care using the existing infrastructure can be used in the prediction and early diagnosis of preeclampsia.

## Background:

Preeclampsia is a severe health condition that occurs during pregnancy and is diagnosed at a gestation age of 20 weeks by the manifestation of high blood pressure and proteinuria. The development of hypertension is a significant risk factor in maternal and fetal morbidity and mortality globally, with the preeclamptic women' populations such from 3% to 8% of the countries [1]. WHO revealed that preeclampsia was the second most common cause of maternal death worldwide and in less developed countries such as India, it is most of the many causes of maternal death [2]. The prevalence of hypertensive disorders of pregnancy in India has been reported to be 7.8% with preeclamptic women accounting for 5.4% of them which is a significant public health concern in the country recently [3]. Although the exact mechanisms responsible for preeclampsia are not fully understood, it is thought that these at least partially relate to abnormal placentation occurring during the early stages of pregnancy and subsequently the onset of systemic inflammation and endothelial dysfunction [4]. An increased platelet count might result from unregulated activation and use of the maternal circulation of platelets through the culmination of lower counts of platelet and increased Mean Platelet Volume (MPV) [5]. It has been proven that MPV, which represents platelet size and function, is higher in pregnant women with preeclampsia in comparison to those who have normal pregnancies [6]. Furthermore, Platelet Distribution Width (PDW), representing the variability in the size of platelets and Plateletcrit (PCT), representing the total mass of platelets, are known to be affected in preeclampsia and this alteration can be used to assess disease severity [7]. As a marker of systemic inflammation, the Platelet/Lymphocyte Ratio has emerged and gained significance as a possible predictor of the severity of preeclampsia. According to various studies, PLR values are significantly lower among pregnant females diagnosed with severe preeclampsia in comparison with those found in milder variants of preeclampsia or compared with normal pregnancies [8- 9]. Further, non-invasive methods such as uterine artery Doppler assessment have also emerged to predict pregnancy complications associated with utero placental insufficiency. This ability helps healthcare providers evaluate the placental perfusion and directs management strategies in high-risk pregnancies [10]. Despite advancements in our understanding of preeclampsia, uncertainties remain in its diagnosis, screening and management. There is a pressing need for a simple, widely accessible and affordable test that can identify women at risk for preeclampsia before the onset of symptoms. Early detection would enable healthcare providers to closely monitor at-risk women, improving prenatal care and outcomes for both mothers and their foetuses. Given the high incidence of hypertensive disorders of pregnancy and the preventable nature of related maternal deaths, early intervention through improved screening and monitoring is critical [3]. Therefore, it is of interest to determine the relationship of platelet count indices, including mean platelet volume, platelet distribution width, platelet crit and platelet-to-lymphocyte ratio, with the severity of preeclampsia.

## Methodology:

This prospective observational study was conducted among antenatal women with gestational age ranging from 11 to 14 weeks, attending the Antenatal Outpatient Department of the Department of Obstetrics and Gynaecology at SN Medical College, Agra, Uttar Pradesh, India, From April 2021 to September 2022. A total of 205 women of approximately 320 screened antenatal women were selected for the study after Institutional Ethical clearance (Ref. No. IEC/2021/25) and screening based on the predefined inclusion criteria. After the exclusion criteria were applied, 8 women left during the study period, while 17 women had spontaneous miscarriage or termination unrelated to preeclampsia. Thus, a total of 180 were included in the study. The Inclusion criteria for this study included pregnant women in the first trimester (11-14 weeks of gestation) attending antenatal clinic. Women who denied consent to the research, or those presenting with chronic kidney disease, diabetes mellitus, chronic hypertension, thyroid diseases, autoimmune diseases, cardiovascular disorders, or signs of active infection were excluded from the study. A 2mL blood sample was taken from each participant in aseptic conditions using vials containing ethylene diamine tetra acetic acid (EDTA). Samples were analysed at the Department of Pathology at SN Medical College, Agra, Uttar Pradesh, India, using the Automated Counter Sysmex XN1000. Among the platelet indices that were evaluated included platelet count (PC), mean platelet volume (MPV), platelet distribution width (PDW), plateletcrit (PCT) and platelet/lymphocyte ratio (PLR). Abnormal platelet indices were defined as a count <150 x10^9/L, plateletcrit <0.10%, mean platelet volume >10 fL, platelet distribution width >12% and platelet/lymphocyte ratio <132. Furthermore, Doppler evaluations of the uterine artery were carried out at the Department of Radiodiagnosis at SN Medical College, Agra Uttar Pradesh, India. The Pulsatility Index (PI), Resistivity Index (RI) and Systolic/Diastolic (S/D) Ratio were measured; abnormal values were established based on other studies: a mean PI > 1.5 (>95th percentile), a mean RI > 0.7 (>95th percentile) and a mean S/D Ratio > 2.5 (>95th percentile). Detailed clinical histories of each participant were recorded, including age, gravid a, parity, gestational age, socio-economic status, education, personal habits and any history of chronic medical illness. A complete obstetric history, in regard to history of previous childbirths and modes of delivery and time of deliveries, was documented. A menstrual history was taken for the determination of gestational age. The clinical examination included weighing, measuring height and blood pressure. Additionally, there were examinations for pallor, thyroid function and breast examination. Systemic assessments for neurological systems, cardiovascular and respiratory systems were also conducted to eliminate medical or surgical conditions.

Routine investigations included blood grouping and Rh factor typing, a CBC, detection of HBsAg and HIV, measurements of fasting and postprandial blood glucose, assessments of liver function tests (serum bilirubin, SGPT and serum ALP), kidney function tests (blood urea and serum creatinine), urinalysis and ultrasound studies for the well-being of the fetus. Investigatory interventions involved platelet indices and uterine artery Doppler ultrasound for evaluating the risk and progression of preeclampsia.

## Statistical analysis:

The data is expressed as mean and standard deviation (SD) or percentage as appropriate. Quantitative data was measured using Mean ± Standard Deviation. All the categorical variables were expressed using frequency measures; comparison between groups was made by using chi square test. Sensitivity, specificity, positive predictive value (PPV) and negative predictive value (NPV) were used for diagnosing of preeclampsia by uterine artery Doppler and platelet indices. For determination of sensitivity and specificity, receiver operating curve (ROC) analysis was performed. The p-value <0.05 was considered as significant. The statistical analysis was done using SPSS 24.0 version (Chicago, Inc., USA) windows software.

## Follow-up:

The selected patients were taken to attend routine antenatal visits like any other antenatal women. To be more specific, follow-ups were performed at 22-24 weeks for the primary assessment, at 31-34 weeks for the secondary assessment and after 36 weeks till the time of delivery for the tertiary assessment, where platelet indices and uterine artery Doppler studies were conducted. All participants in the study were followed up until delivery and all data on maternal blood pressure and maternal/neonatal adverse outcome events were collected. The frequency of follow-up was higher for patients with high risk factors. The monitoring became more frequent and vigorous in patients who developed preeclampsia, keeping watch over blood pressure with frequent visits and additional ultrasounds coupled with colour Doppler imaging.

## Results:

Total number of patients who came under the study was 180 in numbers; out of them, 19 (10.60%) developed preeclampsia. Among them, 6 patients were diagnosed with the severe form of preeclampsia, *i.e.,* 31.6% and 13 patients were reported to have had the non-severe form of the condition, *i.e.,* 68.4%. As for the cases of preeclampsia, 57.9% were Primigravida, so null parity is a major risk factor in preeclampsia in Table 1.

The correlation of uterine artery Doppler indices between the groups with preeclampsia and those without, the mean pulsatility index was significantly high in the group with preeclampsia (1.61 ± 0.31) compared to the non-preeclampsia group (1.01 ± 0.21) and there is a statistical significance at P < 0.05. The total resistivity index in the preeclampsia group was recorded to be 0.623 ± 0.121, whereas in the group of patients diagnosed as having non-preeclampsia it was 0.569 ± 0.092; again, this was not statistically significant (P > 0.05). A similar non-statistical difference was noted in the mean S/D ratio, which for the preeclampsia group, was recorded as 1.84 ± 0.42 and in the non-preeclampsia was 1.61 ± 0.342 (P > 0.05) (Table 2).

Besides Doppler indices, the study analysed the association of platelet indices among the groups categorized according to PE and non-PE. The mean count of platelets (x10^9^/L) in the PE group was significantly low, that is, 176.34 ± 11.71 as compared to the one found in the non-PE group, which was 215.07 ± 13.81 and illustrated statistical significance, P < 0.05. In the PE cohort, the MPV was significantly high at 11.61 ± 0.32 compared to the non-PE cohort at 9.74 ± 0.24, with this variation significant at P < 0.05. On the other hand, there was no statistical difference between the two groups as far as PDW and PCT were concerned (P > 0.05). Moreover, the PLR was significantly lower in the PE group (121.59 ± 3.14) than in the non-PE group (134.01 ± 7.28) and the P-value was 0.018 (P < 0.05).3.2 (Table 3).

The combined platelet indices and uterine artery Doppler indices were finally evaluated in correlation with preeclampsia. The abnormal indices were identified in 17 cases (89.47%) of the preeclampsia group, while normal results were obtained in only 2 cases (10.53%). Six cases had false positive results for combined indices in the normotensive group, while 155 were true negatives. The above combination of indices was found to be statistically significant with a (P = 0.007). The combined indices had a sensitivity of 89.47% with a specificity of 96.27%. PPV was 73.91% and NPV was 98.73%, at a 95% CI (Table 4 and Table 5).

## Discussion:

The present study was conducted to evaluate the clinical potential of platelet indices in addition to uterine artery Doppler parameters for the prediction of preeclampsia and its severity. Our results clearly show marked variations between the groups in both platelet indices and Doppler measurements, pointing to their potentials in use as predictive tools in the early identification and management of cases. Agreement with previous studies is found wherein the mean platelet count was significantly lower in the pre-eclamptic group (176.34 ± 11.71) compared to the normotensive group (215.07 ± 13.81), thereby affirming that thrombocytopenia is a feature commonly encountered in preeclampsia [11, 12]. Furthermore, elevated mean platelet volume in pre-eclamptic patients (11.61 ± 0.32) aligns with other studies suggesting that larger, more reactive platelets play a role in the pathophysiology of the condition. A notable finding was the significant decrease in the platelet/lymphocyte ratio (PLR) in pre-eclamptic patients (121.59 ± 3.14) compared to normotensive individuals (134.01 ± 7.28), a marker that has been linked to heightened inflammatory status in preeclampsia [12, 13-14]. Together, these indices demonstrated high sensitivity (89.47%) and specificity (96.27%) for predicting preeclampsia, underscoring their utility as early diagnostic markers. The uterine artery Doppler parameters are in keeping with the literature; it showed that the preeclamptic group has a more elevated PI (1.61 ± 0.31) than in the normotensive group (1.01 ± 0.21). Impaired placental perfusion has also been well-documented Doppler indices, so this study affirms the clinical relevance of PI in predicting outcome [13, 15]. Platelet indices can serve as reliable markers for predicting the severity of preeclampsia [16]. Notably, combining platelet indices and Doppler measurements improved predictive accuracy for preeclampsia (P = 0.007), suggesting that integration of these methods might also improve early detection and thereby facilitate timely interventions to reduce associated maternal and fetal morbidity. Some limitations of our study should be pointed out. Its small sample size and single-centre design reduce its generalizability and its cross-sectional approach forbids any inferences to causality. Other confounding variables such as coexistent medical conditions were not controlled and the inherent variability of Doppler and platelet measurements may confound consistency. Multicentre, longitudinal studies, with standardized measurement processes, larger and more diverse samples are necessary to confirm these observations. Not with standing these limitations, our study supports the potential role of platelet indices and uterine artery Doppler assessment in routine prenatal care as a relatively inexpensive approach to screening for preeclampsia. Such markers may prove useful in improving maternal-fetal outcomes by enabling their early diagnosis and intervention with further research.

## Conclusion:

This study highlights a strong potential in the integration of platelet indices with uterine artery Doppler parameters in the early prediction of preeclampsia. Such integration may enhance antenatal care through timely intervention, thus improving maternal and fetal outcomes. Their incorporation into routine screening may thus significantly reduce the global burden of preeclampsia.

## Figures and Tables

**Table 1:** Demographical distribution of case with and without preeclampsia

**Variables**	**Cases without PE (n=161)**	**Cases with PE (n=19)**	**P- Value**
Age in years	24.82 ± 4.22	24.66 ± 4.168	0.8418
Socioeconomic status (class V)	58% (93/161)	62% (12/19)	0.5663
Educational status (Illiterate)	60% (97/161)	56% (11/19)	0.8394
Educational status (literate)	40% (64/161)	44% (8/19)	0.843
Primigravida	43.9% (70/161)	57.9% (11/19)	0.0972
BMI (kg/m^2^)	23.2 ± 1.6	24.7 ± 1.22	0.278
Residence (rural)	58.89% (95/161)	56.2% (11/19)	0.7873
Residence (urban)	41.11% (66/161)	43.8% (8/19)	0.926

**Table 2:** Sensitivity and specificity of uterine artery doppler indices for predicting preeclampsia

**Index**	**Sensitivity (%)**	**Specificity (%)**	**Positive Predictive Value (PPV, %)**	**Negative Predictive Value (NPV, %)**
Pulsatility Index (PI)	86.67	92.31	70.32	66.67
Resistivity Index (RI)	53.85	92.31	54.29	66.67
S/D Ratio	50	76.92	30	62.5

**Table 3:** Sensitivity and specificity of platelet indices for predicting preeclampsia

**Index**	**Sensitivity (%)**	**Specificity (%)**	**Positive Predictive Value (PPV, %)**	**Negative Predictive Value (NPV, %)**
Platelet Count	66.67	77.69	63.23	73.33
Plateletcrit (PCT)	63.87	48.32	65.32	72.22
Mean Platelet Volume (MPV)	66.67	99.32	99.21	72.22
Platelet Distribution Width (PDW)	33.33	84.62	33.33	68.75
Platelet Lymphocyte Ratio (PLR)	84.53	85.37	71.38	73.63

**Table 4:** Correlation of Combined platelets indices and uterine artery Doppler indices with preeclampsia

	**Preeclampsia (n=19)**		**Normotensive (n=161)**		**Total**	**p-value**
	N	%	N	%		
Abnormal	17	89.47%	6	3.72%	23	0.007
Normal	2	10.53%	155	96.28%	157	

**Table 5:** Diagnostic performance of combined platelet and uterine artery Doppler indices

**Statistic**	**Value**	**95% CI**
Sensitivity	89.47%	66.86% to 98.70%
Specificity	96.27%	92.07% to 98.62%
Positive predictive value (PPV)	73.91%	56.00% to 86.31%
Negative predictive value (NPV)	98.73%	95.43% to 99.65%
